# External validation of a prediction model for disability and pain after lumbar disc herniation surgery: a prospective international registry-based cohort study

**DOI:** 10.2340/17453674.2025.44251

**Published:** 2025-07-07

**Authors:** Allan ABBOTT, Casper Friis PEDERSEN, Henrik HEDEVIK, Catharina PARAI, Martin A GOROSITO, Mikkel ANDERSEN, Tor INGEBRIGTSEN, Tore K SOLBERG, Margreth GROTLE, Bjørnar BERG

**Affiliations:** 1 Unit of Physiotherapy, Department of Health, Medicine and Caring Sciences, Linköping University, Linköping, Sweden; 2 Department of Orthopedics, Linköping University Hospital, Linköping, Sweden; 3 University of Southern Denmark, Center for Spine Surgery and Research, Spine Center of Southern Denmark, Lillebaelt Hospital, Kolding, Denmark; 4 Department of Orthopedics, Sahlgrenska University Hospital, Gothenburg, Sweden; 5 The Sahlgrenska Academy, Gothenburg University, Gothenburg, Sweden; 6 Centre for Intelligent Musculoskeletal Health, Faculty of Health Sciences, Oslo Metropolitan University, Oslo, Norway; 7 Department of Computer Science, Oslo Metropolitan University, Oslo Norway; 8 Department of Neurosurgery and the Norwegian Registry for Spine Surgery, The University Hospital of North Norway, Tromsø, Norway; 9 Department of Clinical Medicine, Faculty of Health Sciences, UiT The Arctic University of Norway, Tromsø, Norway; 10 Department of Research and Innovation, Division of Clinical Neuroscience, Oslo University Hospital, Oslo, Norway

## Abstract

**Background and purpose:**

We aimed to externally validate machine learning models developed in Norway by evaluating their predictive outcome of disability and pain 12 months after lumbar disc herniation surgery in a Swedish and Danish cohort.

**Methods:**

Data was extracted for patients undergoing microdiscectomy or open discectomy for lumbar disc herniation in the NORspine, SweSpine and DaneSpine national registries. Outcome of interest was changes in Oswestry disability index (ODI) (≥ 22 points), Numeric Rating Scale (NRS) for back pain (≥ 2 points), and NRS for leg pain (≥ 4 points). Model performance was evaluated by discrimination (C-statistic), calibration, overall fit, and net benefit.

**Results:**

For the ODI model, the NORspine cohort included 22,529 patients, the SweSpine cohort included 10,129 patients, and DaneSpine 5,670 patients. The ODI model’s C-statistic varied between 0.76 and 0.81 and calibration slope point estimates varied between 0.84 and 0.99. The C-statistic for NRS back pain varied between 0.70 and 0.76, and calibration slopes varied between 0.79 and 1.03. The C-statistic for NRS leg pain varied between 0.71 and 0.74, and calibration slopes varied between 0.90 and 1.02. There was acceptable overall fit and calibration metrics with minor–modest but explainable heterogeneity observed in the calibration plots. Decision curve analyses displayed clear potential net benefit in treatment in accordance with the prediction models compared with treating all patients or none.

**Conclusion:**

Predictive performance of machine learning models for treatment success/non-success in disability and pain at 12 months post-surgery for lumbar disc herniation showed acceptable discrimination ability, calibration, overall fit, and net benefit reproducible in similar international contexts. Future clinical impact studies are required.

Low back pain (LBP) and radiculopathy are leading causes of healthcare seeking and work absence imposing a socioeconomic burden on society [1,2]. After adequate time for natural course recovery and interventions in primary care [3], persistent activity-limiting pain correlating with intervertebral disc herniation symptomology and pathology confirmed on magnetic resonance imaging may indicate lumbar disc herniation surgery [4]. Annual surgical incidence for symptomatic lumbar disc herniation exhibits variation between countries with similar healthcare systems despite similar patient characteristics and outcomes. Sweden has a rate of 29 surgeries/100,000 compared with 46/100,000 in Denmark, and 58/100,000 in Norway [5]. The identification of patients with an elevated likelihood of favorable long-term surgical outcomes has the potential to enhance overall health with significant cost savings for society [6].

In Scandinavia, population-based registries with near-complete national coverage for spinal surgeries faithfully mirror real-world clinical practice and include a broad range of presurgical variables aiding prognostic modeling of outcomes [7-9]. Machine learning prediction models have recently been developed and internally validated based on the Norwegian Registry for Spine Surgery (NORspine), with enhanced discriminative performance for success/non-success in disability and pain outcomes 12 months after lumbar disc herniation surgery [9].

We aimed to externally validate the NORspine models for predicting disability and pain outcomes 12 months after lumbar disc herniation surgery based on similar data from Swedish and Danish national spine surgery registries (SweSpine and DaneSpine).

## Methods

### Design

This study has an external validation design applying the methodological framework described by Riley and colleagues [10] to evaluate the NORspine models for predicting disability and pain outcomes 12 months after lumbar disc herniation surgery [9].

This study was reported in line with the Transparent reporting of multivariable prediction models for Individual Prognosis or Diagnosis + Artificial Intelligence (TRIPOD+AI) statement [11] and was registered in ClinicalTrials.org (NCT05745129).

### Data source and patient population

#### Development cohort

NORspine is a clinical registry for degenerative spine surgeries established in Norway in 2007. It has had a national coverage of 100% (40/40 clinics) since 2016, surgery registration completeness of 81%, and 12-month follow-up rate averaging 72% for all spinal surgeries nationally [12]. In our study, patients in the NORspine registry who had a lumbar discectomy between January 1, 2007, and May 31, 2021, were identified and assessed for eligibility. Surgeries repeated more than 90 days after the initial procedure were recorded as new cases, while those within 90 days were considered complications and not logged as new cases. Patients undergoing surgery due to cauda equina syndrome were excluded from the study. The registry excludes patients undergoing surgery for fractures, trauma, infection, or cancer.

#### External validation cohorts

The SweSpine registry was initiated in 1998 and encompasses surgically treated patients with degenerative spinal conditions. National coverage stands at 98% (46/47 clinics), surgery registration completeness of 86%, and 12-month follow-up rates averaging 70% for all spinal surgeries nationally [13]. For our study, all patients enrolled in the SweSpine registry who underwent lumbar discectomy between January 1, 2016, and December 31, 2022, were identified and screened for eligibility. The reason for not including data from 1998–2015 is explained by the fact that several variables in the NorSpine model were not collected in the SweSpine registry during these years. Surgeries repeated more than 90 days after the initial procedure were recorded as new cases, while those within 90 days were considered complications and not logged as new cases. Additionally, patients who underwent surgical procedures for cauda equina syndrome, metastases, spondylodiscitis, and epidural abscess were excluded. The registry excludes patients undergoing surgery due to primary tumors, fractures, or other trauma. The same inclusion and exclusion criteria were applied for all patients enrolled in the Spine Center of Southern Denmark cohort in DaneSpine between August 1, 2009, and December 31, 2022. This cohort from 1 of 17 clinics providing registrations to DaneSpine was utilized because it provides the largest sample of lumbar discectomy surgeries in the registry (20% of the national coverage) with consistently excellent surgery registration completeness (96%) and averaging 88% 12-month follow-up rate for all spinal surgeries [14].

The data collection process for the NORspine, SweSpine, and DaneSpine registries starts with patient-reported questionnaires at the time of surgical admission (baseline). Surgeons record information related to diagnosis and surgical procedures using a standardized form. At 12 months postoperatively, follow-up questionnaires containing patient-reported outcomes are distributed to the patients via mail/email.

### Outcomes

The study outcomes assessed patient improvements in Oswestry Disability Index (ODI) [15] and the numeric rating scale (NRS) [16] for back pain and leg pain over a 12-month period. The ODI, a 10-item score ranging from 0 (no disability) to 100 (maximum disability), captures limitations in daily activities in relation to LBP. The NRS quantifies pain intensity on an 11-point scale, with 0 indicating no pain and 10 representing the most severe pain imaginable. Treatment success was operationalized based on the event of a minimal clinically important difference of ODI ≥ 22 points; NRS back pain ≥ 2 points; and NRS leg pain ≥ 4 points improvement from baseline to 1 year [9].

### Predictors

A total of 20 preoperative predictors covering patient demographic characteristics, comorbidity, clinical characteristics, analgesics use, and type of operation included in the NORspine database could be matched to variables in the SweSpine and DaneSpine databases. Variables previously included in the development of the NORspine model, specifically unresolved claim issue, paresis grade, marital status, native speaker, and educational level could not be included in the current study due to these variables not being collected in either the SweSpine or DaneSpine cohorts. To accommodate this limitation, a reduced version of the NORspine model was retrained for validation.

### Sample size

Sample size calculation for external validation was conducted based on anticipated non-successful outcome prevalence = 33% and C-statistic = 0.81. We assumed the linear predictor would be normally distributed with a mean of 0.96, standard deviation 1.54, targeting a 95% confidence interval (CI) width of 0.2 for the calibration slope and 0.1 for the C-statistic [9]. This resulted in a minimum sample size of 1,939 (640 non-successful events) for external validation. Sample size in the validation cohorts far exceeds this sample size calculation [17]. The pmvalsampsize package in Stata (StataCorp LLC, College Station, TX, USA) was used for the calculation.

### Data cleaning and quality checks

Integrity of the NORspine, SweSpine, and DaneSpine registries’ data is routinely evaluated identifying any systematic or random error in data recording [12-14]. Additional verifications of data integrity were executed during data cleaning for this study, encompassing examination for duplicate records, outliers, incomplete data, and variables missing in a systematic manner.

### Statistics

Analyses were conducted from January–August 2024. Descriptive statistical methods were utilized for baseline characteristics. To address missing baseline and outcome data, which was presumed to be missing at random, multiple imputation by chained equations was employed, resulting in 50 sets of imputed data in each cohort. The imputation model included all predictors and outcome variables and was conducted separately for the development and the 2 validation cohorts. The number of imputations (50) was determined based on the proportion of individuals with missing data. The consistency of imputations was verified by contrasting the distributions of the imputed values with those of the complete dataset. Subsequently, the measures of predictive accuracy were calculated for each set of imputed data, prior to their amalgamation based on Rubin’s rule [18].

The analyses incorporated all available potential predictor variables, not performing any form of feature selection to preserve the integrity of prognostic data. The trained model objects from the NORspine cohort were applied directly to the external validation cohorts without re-fitting. Continuous variables were normalized through min–max scaling and categorical variables one-hot encoded. The continuous variables remained unaltered to maintain their prognostic value. Optimization of hyperparameters in the training and development of the NORspine model was conducted through systematic grid search complemented by 5-fold cross-validation (Table S1 in Supplementary data). An extreme gradient boosting supervised machine learning algorithm (XGBoost; https://xgboost.ai/) used in training and development of the NORspine model and its hyperparameters were applied in the external validation. The XGBoost algorithm was utilized because it was the optimal algorithm for predicting each specific outcome in the NORspine model development [9]. The NORspine model’s performance to distinguish between outcomes was evaluated on the SweSpine and DaneSpine datasets, with the C-statistic including CI. The interpretation of these values is as follows: 0.5 = no, > 0.5 = poor, ≥ 0.6 = fair, ≥ 0.7 = acceptable, ≥ 0.8 = excellent, ≥ 0.9 = outstanding success/non-success outcome discrimination. Calibration slopes (1 = perfect agreement), calibration-in-the-large (CITL, 0 = perfect agreement), and Brier score overall fit (0 = perfect predictive accuracy) were also assessed.

Calibration plots were created to compare agreement between observed and the model’s predicted probabilities of successful outcome events, using the pmcalplot function in Stata. Points below the diagonal agreement line indicate that the model’s successful outcome event estimates are too high compared with observed events while points above the line indicate that the model’s successful outcome estimates are too low compared with observed events. These plots were produced for every imputed dataset and examined for consistency across imputations. The clinical utility of the models was assessed through decision curve analysis, which involved comparing potential net-benefit trade-off between the benefits of true positives (successful outcomes) and the potential unnecessary surgeries that may arise from false positives, across a range of threshold probabilities. The models were compared with default strategies of treating all or none. To determine the ranking of importance of predictor variable features within the 3 prediction models, SHAP values were computed. The machine learning algorithms were executed using Python version 3.8.13 (https://www.python.org/downloads/release/python-3813/) and the Scikit-learn (https://scikit-learn.org/stable/) library. Stata software was utilized for data cleaning and multiple imputation. For sensitivity analysis, we compared the results from the imputed data with those from complete case analysis for each outcome.

### Ethics, registration, data sharing plan, funding, and disclosures

The NORspine models were developed based on data approved by the ethics committee of the Health Region of South-East Norway (2022/371282). Use of anonymized SweSpine data received ethical approval obtained from the Regional Ethics Committee in Linköping (Approval Number: Dnr2021–04918). Use of anonymized data owned by the Spine Center of Southern Denmark, Kolding and registered in DaneSpine was deemed to be exempt from review under Danish law. All participant registrations in the NORspine, SweSpine, and DaneSpine registries are informed and consent to anonymized data being utilized in research and development projects. NORspine national cohort data is owned by the Norwegian state, SweSpine national cohort data is owned by the Swedish State, and DaneSpine cohort data used in this study is owned by the Spine Center of Southern Denmark. This prevents data sharing outside of this project. The analytic code is available from the authors upon request. The study is a part of the large-scale AID-Spine project funded by the Research Council of Norway (grant 324915). The Research Council of Norway had no role in the design and conduct of the study; collection, management, analysis, and interpretation of the data; preparation, review, or approval of the manuscript; and decision to submit the manuscript for publication. No conflicts of interests were reported by the authors. Complete disclosure of interest forms according to ICMJE are available on the article page, doi: 10.2340/17453674.2025.44251

## Results

### Patients

**Figure 1:**
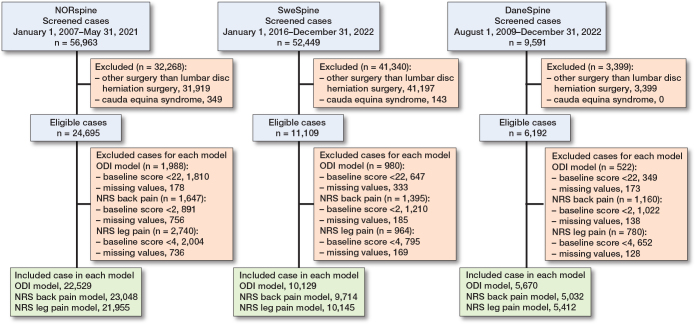
Flow diagram of surgical cases included in the analyses.

The NORspine models were developed and internally validated based on 22,529 disc herniation surgery cases for prediction of ODI outcomes, 23,048 cases for NRS back pain outcomes, and 21,955 cases for NRS leg pain outcomes (Figure 1). Cohorts were screened from 56,963 surgical cases for varying diagnoses in the registry. The cohort for ODI outcomes had a mean age of 47.0 (SD 14.0) years and consisted of 12,854 (57%) males and 9,675 females (43%). The SweSpine cohort is based on 10,129 disc herniation surgery cases for the model predicting ODI outcomes, 9,714 cases for NRS back pain outcomes, and 10,145 cases for NRS leg pain outcomes. These cohorts were screened from 52,449 surgical cases in the registry. The cohort for ODI outcomes had a mean age of 44.9 (SD 13.6) years and consisted of 5,574 (55%) males and 4,554 females (45%). The DaneSpine cohort is based on 5,670 disc herniation surgery cases for the model predicting ODI outcomes, 5,032 cases for NRS back pain outcomes, and 5,412 cases for NRS leg pain outcomes. These cohorts were screened from 9,591 surgical cases in the registry. The cohort for ODI outcomes had a mean age of 48.6 (SD 14.3) years and consisted of 2,904 (51%) males and 2,766 (49%) females. Treatment non-success was experienced by 33%, 29%, 38% (ODI), 27%, 31%, 35% (NRS back pain), and 31%, 38%, 33% (NRS leg pain) of the patients in the NORspine, SweSpine, and DaneSpine cohorts respectively. Further characteristics of these cohorts are presented in Table 1.

**Table 1:** Study populations and variables in the models. Values are count (%) unless otherwise specified

Item	Development cohort		Validation cohorts			
	NORspine January 1, 2007–May 31, 2021 n = 22,529 / n = 14,859		SweSpine January 1, 2016–December 31, 2022 n = 10,129 / n = 5,619		DaneSpine August 1, 2009–December 31, 2022 n = 5,670 / n = 3,701	
	Valid	Missing	Valid	Missing	Valid	Missing
ODI (0–100) ≥ 22^a^	9,934 (67)	7,670 (34)	3,974 (71)	4,510 (45)	2,310 (62)	1,969 (35)
Sex		0 (–)		1 (< 0.1)		0 (–)
Male	12,854 (57)		5,574 (55)		2,766 (49)	
Female	9,675 (43)		4,554 (45)		2,904 (51)	
Mean age (SD)	47.0 (14.0)	0 (–)	44.9 (13.6)	16 (0.2%)	48.6 (14.3)	0 (–)
Mean body mass index (SD)	27.0 (4.5)	1,314 (5.8%)	26.6 (4.4)	449 (4.4%)	26.9 (4.4)	0 (–)
Smoking	5,758 (26)	209 (0.9)	913 (9.1)	100 (1.0)	1,766 (31)	0 (–)
Work status		522 (2.3)		12 (0.1)		
Working or student	5,895 (27)		4963 (49)		1,646 (29%)	0 (–)
Age at retirement^b^	2,256 (10)		950 (9.4)		470 (8.3%)	
Sick leave	10,814 (49)		3,812 (38)		3,251 (57%)	
Disability benefit/work						
assessment allowance	3,042 (14)		392 (3.9)		303 (5.3%)	
Anxiety or depression^c^	8,833 (40)	355 (1.6)	6,909 (69)	82 (0.8)	1,804 (32%)	18 (0.3)
Comorbidities		0 (–)		580 (5.7)		0 (–)
0	16,795 (75)		8,412 (88)		5,246 (93%)	
1	3,850 (17)		949 (9.9)		397 (7.0%)	
2	1,349 (6.0)		174 (1.8)		27 (0.5%)	
≥ 3	535 (2.4)		14 (0.1)		0 (–)	
ASA grade (1–5) ≥ 3	1,469 (6.6)	296 (1.3)	461 (4.9)	739 (7.3)	5 (0.1%)	0 (–)
Mean ODI^a^ (SD)	48.6 (17.2)	0 (–)	50.5 (16.7)	0 (–)	49.4 (16.7)	0 (–)
Mean NRS back pain (SD) ^d^	6.6 (2.4)	550 (2.4%)	5.5 (2.7)	165 (1.6%)	5.2 (2.9)	28 (0.5%)
Mean NRS leg pain (SD) ^d^	7.2 (2.1)	533 (2.4%)	7.4 (2.0)	151 (1.5%)	7.1 (2.3)	19 (0.3%)
Mean EQ-5D (−0.594 to 1) (SD)	0.48 (0.21)	807 (3.6%)	0.47 (0.20)	46 (0.5%)	0.29 (0.33)	109 (1.9%)
Mean EQ VAS (0–100) (SD)	43.2 (21.1)	1,203 (5.3%)	43.7 (21.9)	651 (6.4%)	47.4 (25.5)	76 (1.3%)
Back pain duration		801 (3.6)		74 (0.7)		24 (0.4)
< 3 months	4,164 (19)		1,475 (15)		1,135 (20)	
3–12 months	9,745 (45)		5,163 (51)		2,428 (43)	
12–24 months	3,140 (15)		1,528 (15)		681 (12)	
> 24 months	4,679 (22)		1,889 (19)		1,402 (25)	
Leg pain duration		1,004 (4.5)		47 (0.5)		8 (0.1)
< 3 months	5,505 (25)		1,658 (16)		1,485 (26)	
3–12 months	10,750 (50)		5,808 (58)		2,926 (52)	
12–24 months	2,739 (13)		1,444 (14)		633 (11)	
> 24 months	2,531 (12)		1,172 (12)		618 (11)	
Analgesics use		287 (1.3)		73 (0.7)		0 (–)
Monthly/not using	3,943 (18)		932 (9.3)		475 (8.4)	
Weekly/occasionally	2,873 (13)		2,406 (24)		4,273 (75)	
Daily/regularly	15,426 (69)		6,718 (67)		922 (16)	
Previous surgery		153 (0.7)		46 (0.5)		7 (0.1)
0	17,320 (78)		8,760 (87)		4,545 (80)	
1	3,905 (17)		1,043 (10)		1,118 (20)	
≥ 2	1,151 (5.1)		280 (2.8)		0 (–)	
Microdiscectomy	21,088 (94)	0 (–)	5,310 (52)	0 (–)	3,015 (53)	0 (–)
Surgical levels ≥ 2	1,253 (5.6)	0 (–)	475 (4.7)	24 (0.2)	418 (7.4)	0 (–)
Emergency surgery	4,363 (20)	129 (0.6)	381 (3.8)	145 (1.4)	56 (1.0)	0 (–)

ASA: American Society of Anesthesiologists; NRS: Numeric Rating Scale; ODI: Oswestry Disability Index.

a ODI is a 10-item score from 0 (none) to 100 (maximum disability) encompassing limitations in various activities of daily living. Treatment success is defined based on achievement of the minimal important change (≥ 22 points improvement from baseline).

b Individuals receiving retirement/age pension. While the retirement age in Norway, Sweden, and Denmark is 67 years, individuals have the flexibility to decide when they wish to start receiving their retirement pension.

c EQ-5D questionnaire; 5th item, moderate to severe (3L) or moderate to extreme (5L).

d The NRS measures pain intensity during the last week on an 11-point scale, with 0 representing no pain and 10 the worst imaginable pain.

### Model performance

Model performance estimates are summarized in Table 2. The NORspine development models displayed C-statistic levels of 0.81 (CI 0.80–0.82) indicating excellent level for discriminating success/non-success in ODI, 0.76 (CI 0.75–0.77) for predicting NRS back pain, and 0.74 (CI 0.73–0.75) for predicting NRS leg pain, indicating acceptable level for discriminating success/non-success in these outcomes. The SweSpine validation models displayed C-statistic levels of 0.76 (CI 0.75–0.78) for predicting ODI, 0.72 (CI 0.70–0.73) for predicting NRS back pain, and 0.71 (CI 0.69–0.72) for predicting NRS leg pain, indicating acceptable level for discriminating success/non-success in these outcomes. The DaneSpine validation models displayed C-statistic levels of 0.77 (CI 0.76–0.79) for predicting ODI, 0.70 (CI 0.69–0.72) for predicting NRS back pain, and 0.71 (CI 0.69–0.73) for predicting NRS leg pain, indicating acceptable level for discriminating success/non-success in these outcomes.

**Table 2:** Model predictive performance estimates with 95% confidence intervals pooled across 50 imputed datasets

Item	Development cohort	Validation cohorts	
	NORspine	SweSpine	DaneSpine
ODI, n	22,529	10,129	5,670
C-statistic	0.81 (0.80 to 0.82)	0.76 (0.75 to 0.78)	0.77 (0.76 to 0.79)
Calibration slope	0.99 (0.96 to 1.02)	0.84 (0.78 to 0.90)	0.88 (0.81 to 0.95)
CITL	0.02 (−0.02 to 0.05)	−0.19 (−0.26 to −0.12)	−0.70 (−0.78 to −0.62)
Brier score	0.16 (0.16 to 0.17)	0.17 (0.17 to 0.17)	0.20 (0.20 to 0.21)
NRS back pain, n	23,048	9,714	5,032
C-statistic	0.76 (0.75 to 0.77)	0.72 (0.70 to 0.73)	0.70 (0.69 to 0.72)
Calibration slope	1.03 (0.99 to 1.07)	0.88 (0.81 to 0.95)	0.79 (0.71 to 0.87)
CITL	0.02 (−0.02 to 0.05)	0.07 (−0.01 to 0.14)	−0.24 (−0.32 to −0.16)
Brier score	0.17 (0.17 to 0.17)	0.18 (0.18 to 0.19)	0.21 (0.20 to.21)
NRS leg pain, n	21,955	10,145	5,412
C-statistic	0.74 (0.73 to 0.75)	0.71 (0.69 to 0.72)	0.71 (0.69 to 0.73)
Calibration slope	1.02 (0.98 to 1.06)	0.94 (0.86 to 1.01)	0.90 (0.81 to 0.99)
CITL	0.02 (−0.01 to 0.05)	−0.15 (−0.21 to −0.09)	−0.37 (−0.45 to −0.30)
Brier score	0.18 (0.18 to 0.18)	0.18 (0.17 to 0.18)	0.20 (0.20 to 0,21)

CITL: calibration-in-the-large; NRS: Numeric Rating Scale; ODI: Oswestry Disability Index.

**Figure 2:**
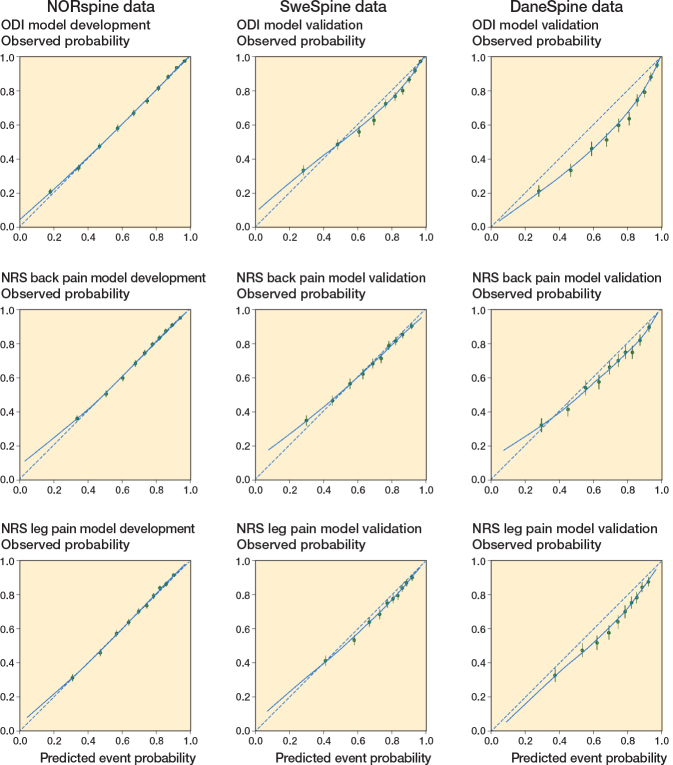
Calibration plots for prediction models in the development and validation cohorts. The dashed diagonal line represents perfect calibration. The solid line is a LOESS-smoothed calibration curve. Points below the dashed line indicate that the model’s successful outcome event estimates are too high compared with observed events; points above the dashed line indicate that the model’s successful outcome estimates are too low compared with observed events.

Calibration of the SweSpine and DaneSpine validation models compared with the NORspine development models is displayed in Figure 2, showing observed probabilities vs the model’s predicted probabilities for successful outcomes. The NORspine models’ CITL are close to 0 and calibration slopes are close to 1, indicating almost perfect agreement between predicted and observed outcomes. The SweSpine NRS back pain model CITL was slightly below the ideal value of 0 but the CI crossed 0, indicating no significant miscalibration with the NORspine model. Other Swespine and DaneSpine validation models had CITL and CI values slightly below the ideal value of 0 showing mild miscalibration except for the DaneSpine ODI model, which showed modest miscalibration. The miscalibration reflects a mild–modest systematic bias overestimating average predictions of successful outcomes compared with the observed probabilities from the models in the external validation cohorts (see Table 2).

Calibration slopes slightly less than the optimal value 1 suggests underestimation of lower probabilities and overestimation of higher probabilities of successful outcomes but the slope CI for the SweSpine leg pain model crossed 1, suggesting no significant miscalibration. Other Swespine and DaneSpine validation models had calibration slopes CI values slightly below the ideal value of 1, reflecting small systematic bias underestimating lower probabilities and overestimating higher probabilities for successful outcomes compared with the observed probabilities from the models. Overall fit of the prediction models combining calibration and discriminative ability for the binary outcome displayed acceptable Brier scores (see Table 2).

### Feature importance

Like the NORspine model, successful treatment outcomes for ODI in the SweSpine and DaneSpine models showed the most important predictive features to be greater initial score, lower duration of back pain, lower NRS back pain score, lower duration of leg pain, lower anxiety or depressive symptoms, maintained work status, and non-smoking status. These features were similarly important in predicting the NRS scores for both back and leg pain in the NORspine, SweSpine, and DaneSpine models (Figure S1, see Supplementary data).

### Clinical utility

**Figure 3:**
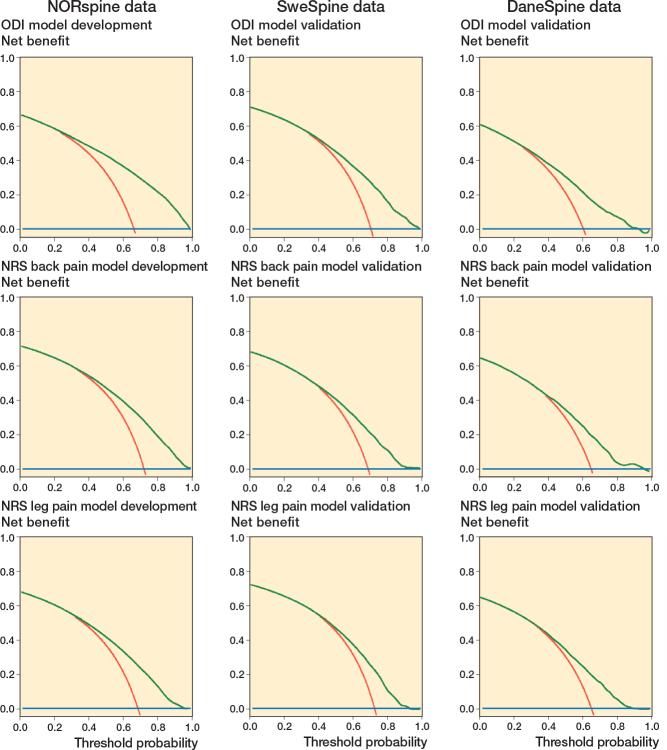
Decision curve analyses for the Oswestry Disability Index, NRS back pain, and NRS leg pain models. Net benefit (y-axis) for binary outcome prediction models across a range of threshold probabilities along the x-axis that define when surgery is warranted. Each line represents 1 prediction result, with red line = treat all, blue line = treat none, and green cline = treat per prediction model.

Decision curve analyses show greater potential net benefit of surgical treatment following the prediction models (NORspine, SweSpine, and DaneSpine) compared with either surgically treating all or no patients with regards to all potential threshold probabilities for success/non-success of ODI, NRS back, and NRS leg pain outcomes (Figure 3).

### Sensitivity analyses

Similar results were found for all 3 models when only surgical cases with complete data were included compared with imputed data (Table S2, see Supplementary data).

## Discussion

In the NORspine development model and its external validation in SweSpine and DaneSpine data cohorts, the outcomes regarding ODI, NRS back pain, and NRS leg pain could be effectively distinguished between patients with successful and non-successful outcomes. This was evidenced by reproducing acceptable–excellent C-statistic levels, minimal to modest miscalibration between observed and predicted successful event probabilities, acceptable overall fit, and clear net benefit.

The development and external validation models in the current study match or surpass the predictive accuracy of previous models in the literature. Nevertheless, the generalizability of most previous models is restricted due to smaller cohorts from individual surgical centers [8,19-27]. Also, few models fulfill the rigor of current methodological recommendations for development and validation, and none have been externally validated showing reproducibility internationally [28].

To determine the clinical utility of the model, decision curve analysis was performed based on the net-benefit trade-off between the benefits of true positives (successful outcomes) and the potential unnecessary surgeries that may arise from false positives, across a range of threshold probabilities. Treatment in accordance with the prediction models in the current study had on average a net benefit of 0.3 at a threshold probability for successful outcome of approximately 70% where the “treat all with surgery” strategy on average had no remaining net benefit. In this case, when compared with treating none or all, treatment in accordance with the prediction model found 30% more patients with successful disability and pain outcomes without conducting any unnecessary surgeries. These results were similar for Norwegian, Swedish, and Danish cohorts. The machine learning models have been integrated into an online calculator (https://huggingface.co/spaces/martingorosito/aidspine_hdsurgery_calculator).

### Strengths and limitations

Despite good relatedness between datasets in the Scandinavian context with similar health and welfare systems, inclusion and exclusion criteria for patients, preoperative timing and measurement of predictors, outcome definitions, event proportions, and low level of missing data in baseline predictive features, some miscalibration should be anticipated due to small differences in population case-mix. These small differences were fewer smokers, fewer receiving sick leave/disability benefits, and more patients experiencing anxiety or depression in the SweSpine cohort. The NORspine cohort had a higher proportion with comorbidities, emergency surgeries, and use of microdiscectomy surgical technique. Comorbidity is answered by the surgeon in NORspine and by the patient in SweSpine and DaneSpine with fewer response options. The DaneSpine cohort had a lower proportion of patients with ASA grade ≥ 3 and fewer with regular analgesic consumption. Variation between countries in rates of emergency surgeries and microdiscectomy surgical technique showed low feature importance in the prediction models, suggesting negligible impact on prediction model miscalibration.

There could also be other potential unmeasured factors influencing case-mix and outcome follow-up. The NORspine, SweSpine, and DaneSpine registries, however, comply with international standards for data collection [29]. Approximately 35–45% of outcomes were missing at 1-year follow-up in the current study’s cohorts but sensitivity analyses for surgical cases with complete data compared with imputed outcomes found similar results. Similar outcomes between respondents and non-respondents to follow-up has been analyzed previously in SweSpine data where educational level below university/college, being born outside the EU, smoking, and comorbidity were variables associated with non-response [30].

In the current study, features with higher SHAP importance included in the original NORspine model development [9], such as educational level and native/non-native speaker, could not be included due to missing data in the DaneSpine cohort. Unresolved claim issue, paresis grade, and marital status were further variables included in the original NORspine model development [9] but had low SHAP importance and are completely missing from SweSpine and DaneSpine cohorts. However, the removal of these 5 variables from NORspine development models in the current study resulted only in a negligibly decreased C-statistic (0.01) compared with the original development models [9].

### Conclusions

Predictive performance of NORspine machine learning models for treatment success/non-success in disability and pain at 12 months post-surgery for lumbar disc herniation was externally validated. The models showed acceptable discrimination ability, calibration, overall fit, and net benefit reproducible in similar international contexts but future clinical impact studies are required.

*In perspective,* further research is required on the practical application and models in clinical settings to see if they lead to a higher percent of successful outcomes for lumbar disc herniation surgery.

### Supplementary data

Tables S1, S2, and Figure S1 are available as supplementary data on the article page, doi: 10.2340/17453674.2025.44251

## Supplementary Material



## Notes

Concept and design: AA, CFP, BB, TI, TS, MG. Acquisition, analysis, or interpretation of data: AA, CFP, BB, HH, CP, MA, TI, TS, MG. Drafting of the manuscript: AA, BB, CP. Critical review of the manuscript for important intellectual content: AA, CFP, BB, HH, CP, MG, MA, TI, TS, MG. Statistical analysis: BB, HH, CFP and had full access to the NORspine, SweSpine and DaneSpine data respectively and are responsible for the integrity of the data. BB takes responsibility for accuracy of the data analysis. Obtained funding: MG. Administrative, technical, or material support: AA, TS, MG. Supervision: AA, TS, MG.

Handling co-editors: Ilkka Helenius and Philippe Wagner

*Acta* thanks Laura Bonnet, Johan Marjamaa, and Mikko S Venäläinen for help with peer review of this manuscript.
