# Supplementary material for: External validation of a prediction model for disability and pain after lumbar disc herniation surgery: a prospective international registry-based cohort study
Source: Acta Orthop. 2025 Jul 7;96:512–20. doi: 10.2340/17453674.2025.44251 (PMC12232440; doi:10.2340/17453674.2025.44251)
Supplement: Supplementary file 1 [file ActaO-96-44251-s1.pdf]

Supplementary Online Content

Table S1. Hyperparameters of machine learning models [a].

Table S2. Machine learning model predictive performance estimates with 95% confidence intervals in complete cases.

Figure S1. Shapley Additive Explanations (SHAP) Summary Plots of Variable Importance for the Oswestry Disability Index, NRS back pain and NRS leg pain models. Predictive features are arranged along the y-axis based on their importance. Each dot represents one prediction result, with the colors indicating high (red) to low (blue) feature values. SHAP values on the x-axis indicate the distribution of the prediction among the features; a positive value contributes to treatment success, while a negative value contributes to non-success.

Table S1. Hyperparameters of machine learning models [a]

			**Chosen value**		
**Model**	**Hyperparameter**	**Searched value**	**ODI**	**NRS back pain**	**NRS leg pain**
XGBoost	Learning rate	0.005, 0.01, 0.1	0.1	0.1	0.1
	Max depth	1, 2, 4, 8, 10	1	1	1
	N of estimators	100, 500, 1,000	1,000	500	500

[a] Hyperparameters were tuned on the development data using 5-fold grid search.
Abbreviations: N = Number; NRS = Numeric Rating Scale; ODI = Oswestry Disability Index

Table S2. Model predictive performance estimates with 95% confidence intervals in complete cases.

	Development cohort	Validation cohorts	
	**NORspine**	**SweSpine**	**DaneSpine**
ODI	n = 11,615	n = 4,249	n = 3,559
C-statistic	0.82 (0.81 to 0.83)	0.77 (0.75 to 0.79)	0.78 (0.77 to 0.80)
PPV	0.80 (0.79 to 0.81)	0.78 (0.77 to 0.79)	0.70 (0.69 to 0.72)
NPV	0.68 (0.66 to 0.69)	0.62 (0.58 to 0.65)	0.73 (0.70 to 0.77)
Calibration slope	0.99 (0.95 to 1.03)	0.86 (0.79 to 0.92)	0.87 (0.80. 0.94)
CITL	0.02 (−0.03 to 0.06)	−0.13 (−0.21 to −0.06)	−0.68 (−0.75 to −0.60)
Brier score	0.16 (0.15 to 0.16)	0.17 (0.16 to 0.17)	0.20 (0.19 to 0.20)
NRS back pain	n = 12,109	n = 3,988	n = 3,278
C-statistic	0.76 (0.75 to 0.77)	0.72 (0.71 to 0.74)	0.71 (0.69 to 0.72)
PPV	0.78 (0.78 to 0.79)	0.75 (0.74 to 0.77)	0.71 (0.69 to 0.73)
NPV	0.62 (0.60 to 0.65)	0.61 (0.57 to 0.64)	0.64 (0.60 to 0.68)
Calibration slope	1.03 (0.98 to 1.08)	0.87 (0.79 to 0.95)	0.76 (0.68 to 0.84)
CITL	0.01 (−0.03 to 0.06)	0.02 (−0.06 to 0.09)	−0.27 (−0.35 to −0.19)
Brier score	0.16 (0.16 to 0.17)	0.19 (0.18 to 0.19)	0.20 (0.20 to 0.21)
NRS leg pain	n = 11,477	n = 4,167	n = 3,564
C-statistic	0.74 (0.74 to 0.75)	0.71 (0.69 to 0.73)	0.72 (0.70 to 0.73)
PPV	0.76 (0.75 to 0.77)	0.76 (0.75 to 0.78)	0.71 (0.70 to 0.73)
NPV	0.63 (0.61 to 0.65)	0.58 (0.53 to 0.64)	0.61 (0.57. 0.66)
Calibration slope	1.03 (0.98 to 1.08)	0.93 (0.83 to 1.02)	0.86 (0.78 to 0.95)
CITL	0.02 (−0.03 to 0.06)	−0.09 (−0.17 to −0.02)	−0.37 (−0.45 to −0.30)
Brier score	0.18 (0.17 to 0.18)	0.17 (0.17 to 0.18)	0.20 (0.19 to 0.21)

Estimates with 95% confidence intervals are pooled across 50 imputed datasets.
Abbreviations: CITL = Calibration-in-the-large; NPV = Negative Predictive Value; NRS =Numeric Rating Scale; ODI= Oswestry Disability Index; PPV = Positive Predictive Value.

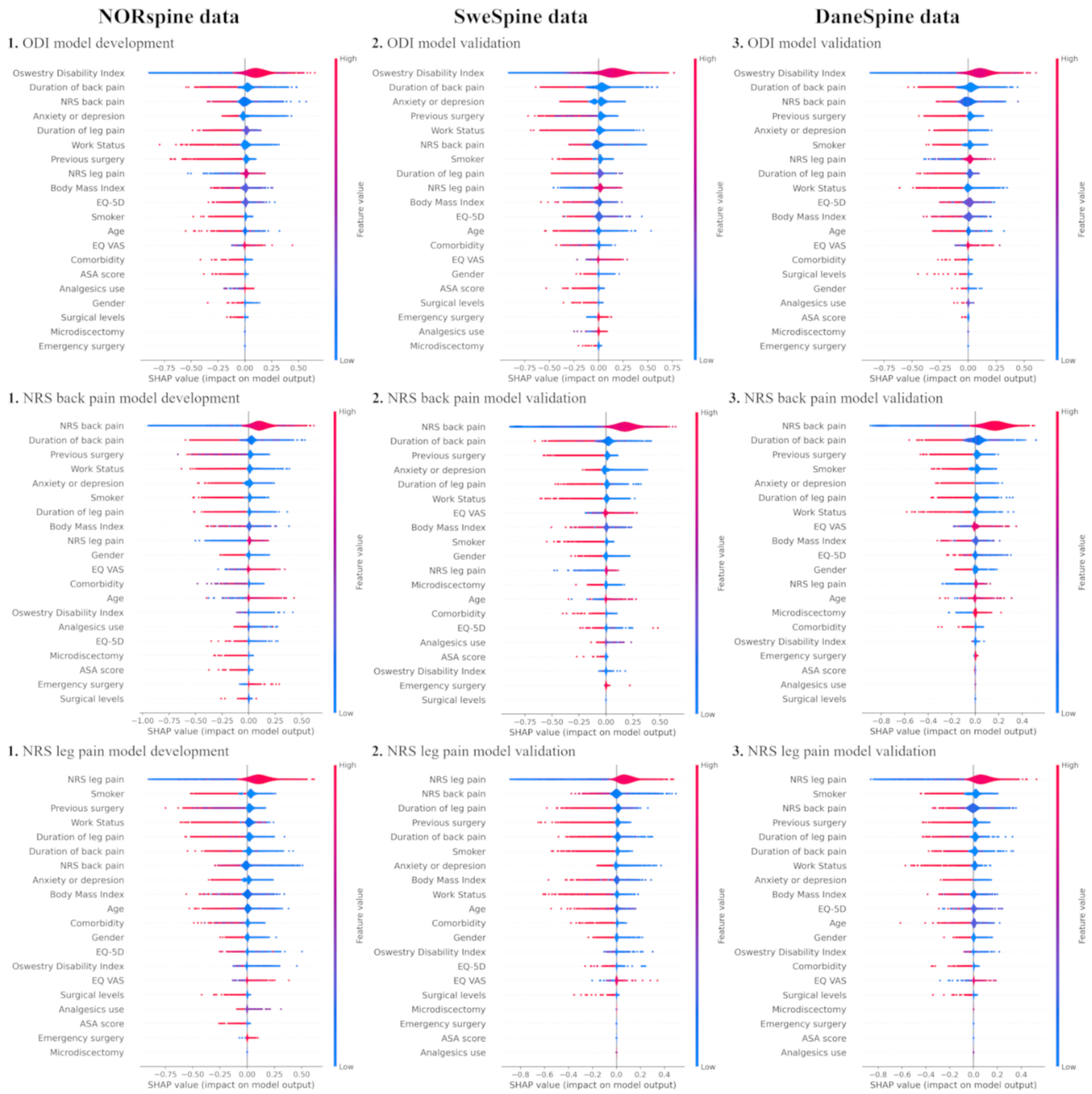

Figure S1. Shapley Additive Explanations (SHAP) Summary Plots of variable importance in the prediction models. Predictive features are arranged along the y-axis based on their importance. Each dot represents one prediction result, with the colors indicating high (red) to low (blue) feature values. SHAP values on the x-axis indicate the distribution of the prediction among the features; a positive value contributes to treatment success, while a negative value contributes to non-success.
